# Compartmentalization and transporter engineering strategies for terpenoid synthesis

**DOI:** 10.1186/s12934-022-01819-z

**Published:** 2022-05-23

**Authors:** Ke Jin, Hongzhi Xia, Yanfeng Liu, Jianghua Li, Guocheng Du, Xueqin Lv, Long Liu

**Affiliations:** 1 Key Laboratory of Carbohydrate Chemistry and Biotechnology, Ministry of Education, Jiangnan University, Wuxi, 214122 China; 2 Science Center for Future Foods, Jiangnan University, Wuxi, 214122 China; 3 Richen Bioengineering Co., Ltd, Nantong, 226000 China

**Keywords:** Terpenoids, Compartmentalization, Transporter, Metabolic engineering

## Abstract

Microbial cell factories for terpenoid synthesis form a less expensive and more environment-friendly approach than chemical synthesis and extraction, and are thus being regarded as mainstream research recently. Organelle compartmentalization for terpenoid synthesis has received much attention from researchers owing to the diverse physiochemical characteristics of organelles. In this review, we first systematically summarized various compartmentalization strategies utilized in terpenoid production, mainly plant terpenoids, which can provide catalytic reactions with sufficient intermediates and a suitable environment, while bypassing competing metabolic pathways. In addition, because of the limited storage capacity of cells, strategies used for the expansion of specific organelle membranes were discussed. Next, transporter engineering strategies to overcome the cytotoxic effects of terpenoid accumulation were analyzed. Finally, we discussed the future perspectives of compartmentalization and transporter engineering strategies, with the hope of providing theoretical guidance for designing and constructing cell factories for the purpose of terpenoid production.

## Introduction

Terpenoids are the largest and most diverse class of natural products, with over 80,000 different structures identified in plants, microorganisms, and marine organisms [1, 2]. All terpenoids are derived from the isoprene (C5) unit isopentenyl diphosphate (IPP) and its isomer dimethylallyl diphosphate (DMAPP). Terpenoids are classified as monoterpenes (C10), sesquiterpenes (C15), diterpenes (C20), triterpenes (C30), tetraterpenes (C40), and polyterpenes (C > 40) based on the number of isoprene units [3]. For instance, menthol (monoterpene) is one of the most important flavoring additives [4], artemisinic acid (sesquiterpene) is a well-known antimalarial drug [5], paclitaxel (diterpene) can be used as an anticancer drug [6], ginsenoside (triterpene) can inhibit the growth of tumor cells [7], and lycopene (tetraterpene) has a high antioxidant potential [8]. Terpenoids have been extensively applied in pharmaceutical and industrial sectors for decades owing to their diverse biological activities and high bioavailability, thereby resulting in their large market demand.

The two primary methods for obtaining plant terpenoids are plant extraction and chemical synthesis [9]. However, because the terpenoid concentration is low in plants and other problems, such as long cycles and environmental dependence on plant culture, scaling up the production of plant extraction is challenging [10, 11]. While the multiple stereocenters of most terpenoids complicate product synthesis and separation in the traditional chemical synthesis [12], this approach is also accompanied by the use of several organic reagents, which can exert a strain on the environment. To avoid the aforementioned problems, the development of microbial cell factories for efficient terpenoid synthesis has emerged as an important research direction for the sustainable production of terpenoids because microbial cell factories provide benefits of low cost, environmental friendliness, and high production efficiency [13, 14].

Terpenoid production by fermentation of microbial chassis has achieved remarkable results in recent years because of the rapid development of metabolic engineering and synthetic biology [13, 15]. Paddon et al., for instance, developed a strain of *Saccharomyces cerevisiae* for high-yielding biological production of artemisinic acid, with a fermentation titer of 25 g/L, which met commercialization, by providing the complete biosynthetic pathway and optimizing the fermentation process [16]. Wang et al. successfully constructed a chassis cell with a high yield of ginsenoside aglycone protopanaxadiol through modular engineering of the mevalonic acid (MVA) pathway and optimizing the P450 enzyme expression levels. The production of ginsenoside Rh2 was found to substantially increased, with 179.3 mg/L in shake flakes and 2.25 g/L in 10-L fed-batch fermentation, when combined with the regulation strategy of increasing glycosylation modification [11]. Currently, some researchers have suggested that in addition to enhancing the synthesis pathway [17, 18], rewriting the central carbon metabolism [19], balancing the competition pathway [20, 21], and heterologously expressing cytochrome P450 enzymes may be effective ways for terpenoid synthesis [22, 23]. However, apart from the traditional metabolic strategies, researchers now found that making full use of organelles in microorganisms may provide new insights to further improve the yield of terpenoid, such as increasing the storage capacity [24, 25].

This review focuses on the strategies for improving storage capacity and compartmentalization regulation for plant terpenoid synthesis using microbial cell factories. The physiological properties of various organelles, in addition to their benefits and drawbacks for compartmentalization, were thoroughly analyzed. The efflux pumps or secretion strategies to export terpenoids were also reported. Finally, the future perspectives and challenges associated with compartmentalization and transporter engineering strategies were thoroughly discussed.

## Compartmentalization strategies for effective synthesis of terpenoids

Eukaryotes can synthesize and store terpenoids via a complete and orderly production line depending on the various organelles and membrane structures they possess such as the endoplasmic reticulum (ER), Golgi complex, lipid droplets (LDs), peroxisomes, mitochondria, and plasma membrane (PM) [26]. However, terpenoid accumulation has the potential to cause cell toxicity, whereas the compartmentalization strategy can not only improve the catalytic efficiency of enzymes but also avoid the harmful effects of toxic substances on cells [26–28]. As a result, studies are being conducted for immobilizing the enzymes required for terpenoid synthesis in various organelle compartments.

### ER engineering

**Fig. 1:**
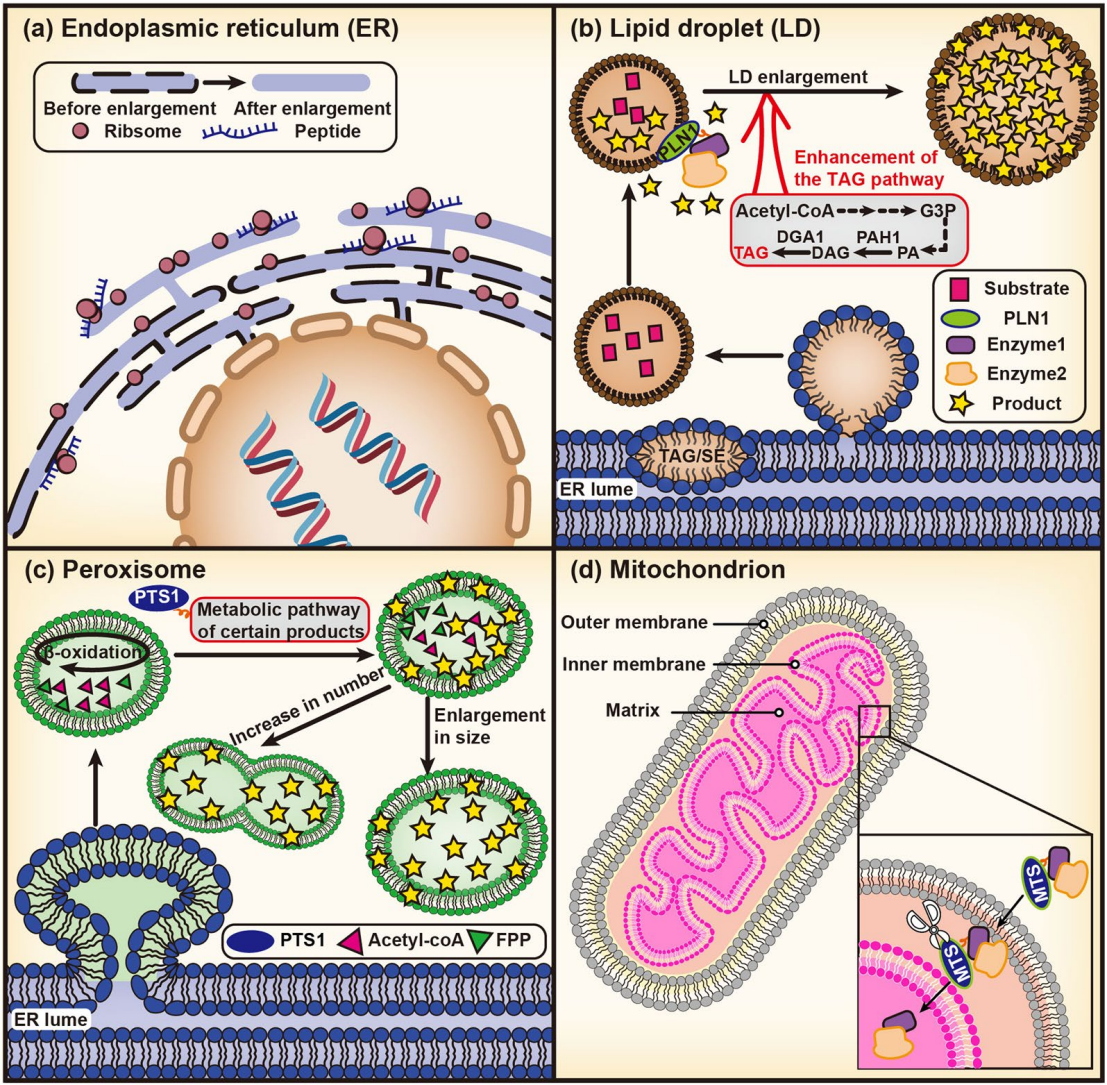
**a** ER, **b** LD, **c** peroxisome and **d** mitochondrion used for compartmentalization strategies. G3P: glycerol-3-phosphate; PA: phosphatidic acid; DAG: diacylglycerol; TAG: triacylglycerol; SE: sterol esters; PLN1: perilipin; PAH1: phosphatidic acid phosphatase; DGA1: diacylglycerol acyltransferase

Other than nucleic acids, ER serves as the base for the synthesis of a series of important substances in cells [29]. ER is speculated to have a crucial role in terpenoid synthesis either in the form of enzyme synthesis and processing or specific ER localization of some enzymes such as key enzymes in the MVA pathway and cytochrome P450 [30]. Thus, ER engineering is a focus of research to improve the efficiency of terpenoid synthesis. Recently, ER engineering has primarily focused on increasing the ER size (Fig. 1a) [31].

After deleting the phosphatidic acid phosphatase-encoding *PAH1* in *S. cerevisiae* and *Yarrowia lipolytica*, a dramatic expansion in ER was observed (Table 1) [32, 33]. The massive proliferation of ER membranes is frequently followed by improvement in their ability to synthesize and fold protein, as well as an increase in protein accumulation levels [34, 35]. For instance, the *S. cerevisiae* strain with disrupted *PAH1* expression could accumulate more *Glycyrrhiza glabra* β-amyrin synthase (GgbAS), and the accumulation of triterpenoids, including oleanane-type sapogenin β-amyrin, and medicagenic-28-O-glucoside, respectively increased by 8-fold and 16-fold in the Δ*pah1* strain compared with the control strain [33]. Notably, *PAH1* knockout tends to reduce the number of LDs [36], and some key enzymes, such as the oxidosqualene cyclases (OSCs), usually require to be positioned in LDs so that they may function normally. As a result, the trade-off between ER and LDs must be considered [37, 38]. Overexpression of the key ER size regulatory factor *INO2* causes ER expansion and a dramatic boost in the yield of squalene and ginsenoside by 128 times and 7 times, respectively [34, 35].

**Table 1:** Terpenoid production using different organelle compartmentalization strategies

Organelle	Yeast species	Products	Titer or yield	Major engineering strategies	References
Endoplasmic reticulum	*S. cerevisiae*	β-amyrin	N.A.	Knock out *PAH1*	[33]
		Aedicagenic-28-O-glucoside	27.1 mg/L	Knock out *PAH1*	[33]
		Artemisinic acid	N.A.	Knock out *PAH1*	[33]
		Squalene	634 mg/L	Overexpression of *INO2*	[35]
		Ginsenoside	12.1 mg/L	Overexpression of *INO2*	[35]
Lipid droplet	*Y. Lipolytica*	Lycopene	16 mg/g	Strengthen the isoprenoid biosynthesis pathway and block the β-oxidation pathway	[47]
		Squalene	731.18 mg/L	Co-overexpression of *tHMG1* and *DGA1*	[53]
	*S. cerevisiae*	Squalene	445.6 mg/L	Co-overexpression of *tHMG1* and *DGA1*	[54]
		Lycopene	2.37 g/L (73.3 mg/g)	Strengthen the TAG pathway and modulate TAG fatty acyl composition	[49]
		Ginsenoside	5 g/L	Target protopanaxadiol synthase to LDs and strengthen the TAG pathway	[44]
		α-amyrin	1107.9 mg/L	Semi-rational design of MdOSC1, strengthen the MVA pathway and overexpress *DGA1*	[48]
Peroxisome	*P. pastoris*	Lycopene	73.9 mg/L	Target heterologous carotenogenic enzymes to peroxisomes	[74]
		α-humulene	3.2 g/L	Introduce the α-humulene synthesis pathway to peroxisomes	[77]
	*S. cerevisiae*	Squalene	11 g/L	Hybridization of the cytoplasm- and peroxisome-engineered strain	[63]
		Geraniol	2.75 mg/L	Deletion of *PEX30* and *PEX32* and introduce the geraniol synthesis pathway into peroxisomes	[72]
			5.5 g/L	Introduce a complete MVA pathway in peroxisomes	[75]
		(R)-(+)-limonene	2.6 g/L	Introduce a complete MVA pathway in peroxisomes	[75]
		Protopanaxadiol	N.A.	Knock out *PEX11*, *PEX34*, and *ATG36*	[73]
		α-humulene	1726.78 mg/L	Introduce the α-humulene biosynthesis pathway into peroxisomes and block the expression of *ERG9*	[76]
		β-Amyrin	2.6 g/L	Introduce the MVA pathway into peroxisomes	[78]
Mitochondrion	*S. cerevisiae*	Valencene	1.5 mg/L	Co-overexpression of *tHMG1*, mitochondrion-targeted heterologous FDP synthase and amorphadiene synthase	[84]
		Amorphadiene	20 mg/L	Co-overexpression of *tHMG1*, mitochondrion-targeted heterologous FDP synthase and amorphadiene synthase	[84]
		Amorpha-4,11-diene	427 mg/L	Introduce the amorpha-4,11-diene biosynthesis pathway to mitochondria	[86]
		Linalool	21 mg/L	Dual mevalonate pathways in mitochondria and cytoplasm	[88]
		Geraniol	43.3 mg/L	Introduce the geraniol biosynthetic pathway into mitochondria	[89]
		Patchoulol	19.24 mg/L	Introduce the DMAPP pathway into mitochondria	[90]
		Isoprene	2527 mg/L	Introduce the complete MVA pathway together with isoprene synthase (ISPS) into mitochondria	[91]
			11.9 g/L	Dual regulation of cytoplasmic and mitochondrial acetyl-CoA utilization	[92]
Plasma membrane	*S. cerevisiae*	β-Ionone	184 mg/L (32 mg/g)	Target the β-carotene cleavage dioxygenase to the membrane	[95]
	*E. coli*	Astaxanthin	N.A.	Target CrtW and CrtZ to the membrane via a GlpF protein	[94]
		Squalene	612 mg/L	Overexpression of *TSR* to expand membrane volume	[93]
		β-carotene	44.2 mg/g DCW	Overexpression of *ALMGS* and *PLSB/PLSC* to increase membrane surface area and enhance membrane synthesis	[24]

### LD engineering

LDs are ER-derived organelles that serve as the primary storage sites for neutral lipids in cells. The LD core is composed of neutral lipids, primarily including triacylglycerols (TAGs) and sterol esters (SEs) [39]. A single-layer phospholipid membrane containing various proteins involved in the biogenesis and function of the organelle surrounds the core [40]. The co-accumulation and co-occurrence of terpenoids and neutral lipids in LDs not only promote further elucidation of the isolation mechanism of the bioactive defense compounds intracellularly, but also provides an opportunity for metabolic engineering and synthetic biology to engineer the high-yield production and storage of terpenoids in the cells with LDs [41–43].

When enzymes and their substrates are compartmentalized, the transformation ability of engineering strains may be extremely low [44]. Anchoring the key enzymes of distinct biosynthetic steps on the surface or inside LDs promotes increased local concentrations of enzymes and hydrophobic substrates, which result in the efficient production of terpenoids. Indeed, the ectopic expression of enzymes in LDs is inextricably linked to the specific localization proteins or corresponding localization peptides, such as PLN1, which is involved in the formation and stability of LDs [45]. The conversion of dammarenediol-II into protopanaxadiol by a normal ER-localized cytochrome P450 enzyme (protopanaxadiol synthase) is a key step in the synthesis pathway of ginsenoside. Given that LDs are the storage organelles of dammarenediol-II, Shi et al. directed protopanaxadiol synthase to LDs using yeast PLN1 protein as the guide protein to obtain a chassis strain. The production of ginsenoside compound K in engineering a chassis strain was 21.8 mg/L/OD, which was nearly 4.4-fold higher than that using the native ER expression strategy [44, 46].

Another key factor in enhancing the ability of cells to synthesize terpenoids is the storage capacity of LDs. Cytosolic LDs are dynamic organelles that vary in size and morphology, and some small LDs can merge to form larger LDs. As the primary factor for TAG synthesis in the oleaginous yeast *Y. lipolytica*, the expression of diacylglycerol acyltransferases (DGATs) influences the size, quantity, and even distribution of LDs [43]. Overexpression of the DGAT gene *YlDGA1* causes *Y. lipolytica* cells to produce smaller but more numerous LDs, whereas the overexpression of *YlDGA2* (also a DGAT gene) results in the production of larger LDs [39]. Furthermore, deletion of *GUT2* and *POX1*–*POX6* in *Y. lipolytica* increases the size of LDs, because the deletion of *GUT2* can prevent the reduction of the glycerol-3-phosphate pool, whereas deletion of the latter cuts peroxisomal β-oxidation short (Fig. 1b) [47].

Using lycopene synthesis as an example, Matthäus et al. found that increased LDs formation by *Y. lipolytica* could improve the storage capacity of cells for lycopene and subsequently improve lycopene synthesis, with an yield of 16 mg/g cell dry weight (CDW) [47]. Similarly, by overexpressing diacylglycerol acyltransferase (*DGA1*) to increase the intracellular storage capacity of *S. cerevisiae*, along with increased expression of key genes of the MVA pathway, the fermentation yield of α-amyrin in the engineering strain was found to be 106 times higher than that in the control strain [48]. Furthermore, the lycopene yield in *S. cerevisiae* strains overexpressing fatty acid desaturase (*OLE1*) and knocking out seipin (*FLD1*), which regulates the size of LDs, reached 70.5 mg/g CDW, which was 25% higher than that of the original high-yield strain [49].

The hydrophobic environment within LDs makes them excellent storage organelles for terpenoids. As a result, LD compartmentalization has recently become the focus of research for improving the yield of terpenoids [38]. As more clarity is gained on the mechanism underlying LD formation, mining other ways to manipulate the number and size of LDs other than enhancing the TAG pathway can potentially provide a new basis for further improvement of the terpenoid yield [50–54].

### Peroxisome engineering

Peroxisomes are the primary organelles enclosed within a single bilayer membrane, which have an important role in cell detoxification. Peroxisomes can be generated using two pathways: “division” and “regeneration.” In case of division, mature peroxisomes divide to produce offspring peroxisomes [55–57]. Regeneration is a relatively complex process that involves three successive processes: budding from the ER membrane to form precursor membrane vesicles, forming the prototype of the peroxidase body, and finally generating a mature peroxidase body [56]. Peroxisomes have long been associated with fatty acid catabolism, particularly in yeast, because they are the only sites where fatty acid β-oxidation occurs, which implies that this organelle can accommodate hydrophobic chemicals [58–61]. Peroxisomes can also simultaneously produce farnesyl diphosphate (FPP), an important metabolic precursor in isoprenoid biosynthesis, which further suggests that peroxisomes are central to terpenoid synthesis [62].

Peroxisomes can be used as subcellular factories or storage depots for various terpenoids, particularly those in non-oleophilic yeasts, such as *S. cerevisiae*, which lack lipid storage space [55, 63–65]. Peroxisome size and number, similar to LDs, can be regulated in a dynamic manner to optimize terpenoid production [66–68]. Modulating the expression of peroxin (PEX) and autophagy (ATG) protein family members, which are responsible for peroxisome biogenesis and pexophagy, respectively in *S. cerevisiae*, may increase the peroxisome proliferation (Fig. 1c) [56, 57, 69–71]. The co-knockout of *PEX30* and *PEX31*, for instance, resulted in a 5.6-fold increase in the peroxisome number [72]. Thus, by improving the tolerance of yeast cells to geraniol and compartmentalizing the geraniol-producing enzymes into peroxisomes, the titer of geraniol was found to increase by 80% [72]. Similar results were also observed in the protopanaxadiol-producing strain [73]. Furthermore, using peroxisome targeting sequence 1, the non-carotenogenic yeast *Pichia pastoris* was able to produce 73.9 mg/L lycopene by targeting heterologous carotene-producing enzymes to peroxisomes [74]. Similar strategies have been used in the biosynthesis of various monoterpenes [75], sesquiterpenes, including α-humulene [76, 77], and triterpenoids, including squalene [63], β-amyrin [78], and protopanaxadiol [73].

### Mitochondrion engineering

Unlike monolayers of LDs and peroxisomes, mitochondria are semi-autonomous organelles surrounded by two layers of membrane, with their inner membrane forming mutliple ridges, which can gradually release energy through respiration to meet the requirements of various cell activities. Because acetyl-CoA is an important precursor for terpenoid synthesis, mitochondria have a much higher acetyl-CoA content (nearly 20–30 times higher) than the cytoplasm, which increases the feasibility of mitochondrial compartmentalization for terpenoid synthesis [79, 80]. However, IPP and DMAPP, which are synthesized from acetyl-CoA via the MVA pathway, are ATP analogs that are strong inhibitors of the mitochondrial respiratory chain. As a result, when the MVA pathway is integrated into the mitochondria, care should be taken to effectively avoid cytotoxicity [81–83].

Rational utilization of FPP pool is a possible strategy for enhancing the production of exogenous terpenoids, and the presence of FPP pools is another advantage of yeast mitochondria [84]. Heterologous FPP synthase and valencene or amorphadiene synthase were targeted into mitochondria using mitochondrial targeting signals (MTSs) and expressed in the *S. cerevisiae* strain with a truncated and deregulated HMG1, resulting in an 8- and 20-fold increase in the production of valencene and amorphadiene, respectively [84]. The selectivity and applicability of MTSs are frequently associated with the range of mitochondrial compartmentalization applications. At present, the most commonly utilized MTS is the pre-sequence of yeast cytochrome c oxidase subunit IV, which can direct various enzymes into the mitochondrial matrix, both in vitro and in vivo [85]. Of note, once the enzyme has been appropriately transposed into mitochondria, the MTS can be cleaved to avoid the effect of fusion expression on enzyme activity (Fig. 1d) [84, 86].

Mining and screening of more efficient MTSs has been conducted to expand the application of mitochondrial compartmentalization engineering for multigene biosynthetic pathways. Based on the mitochondrial proteome, 6 MTSs were screened for the co-localization of α-santalene synthesis pathway, which consists of 10 expression cassettes capable of converting acetyl-CoA to α-santalene, and the results showed that the production of α-santalene increased by 3.7 times in comparison with the control strain [87]. Indeed, as a promising subcellular organelle for compartmentalization engineering, mitochondria merit further investigation by researchers [88–92].

### PM engineering

**Fig. 2:**
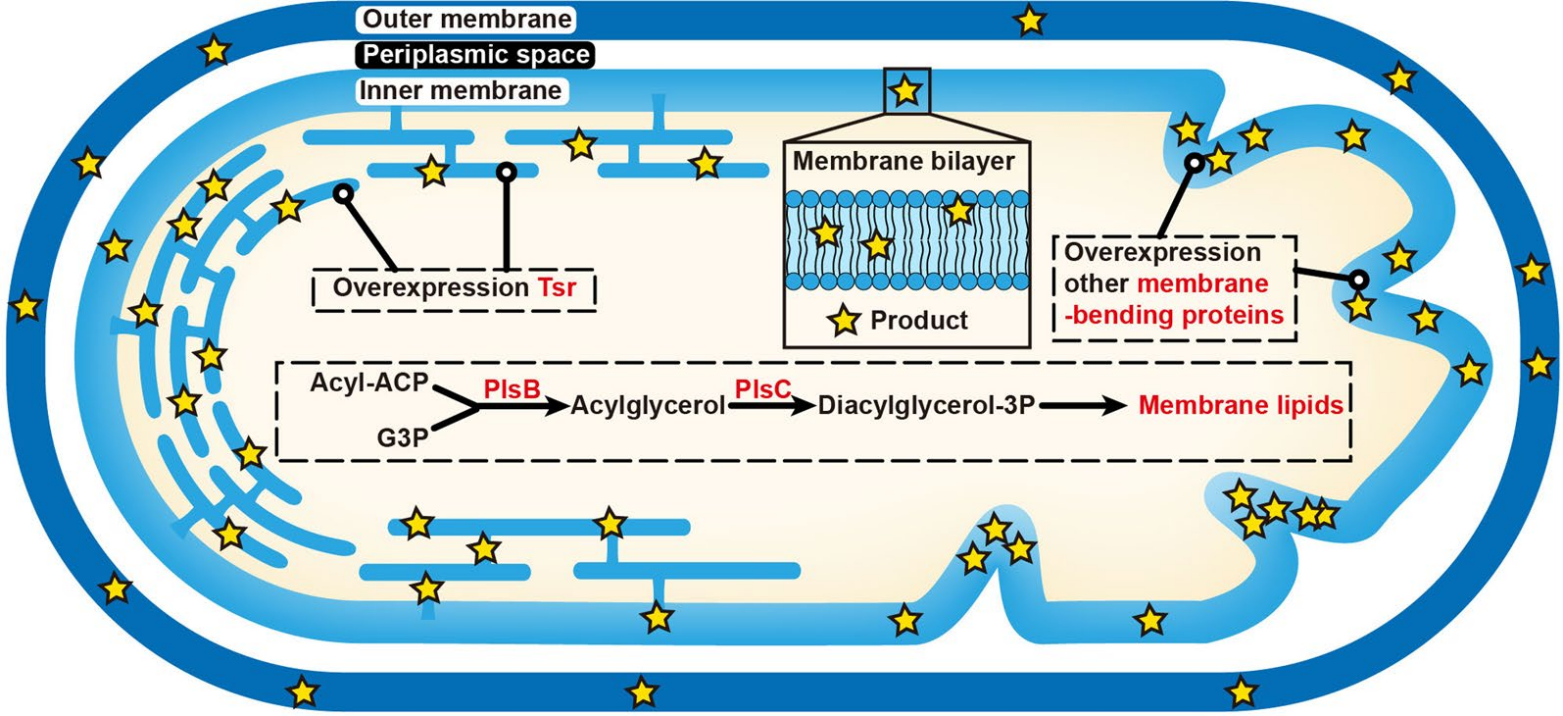
Schematic representation of membrane engineering strategies using *E. coli* as an example. The overexpression of membrane-bending proteins and the enhancement of membrane lipids will lead to membrane expansion for more terpenoid storage. G3P: glycerol-3-phosphate; Diacylglycerol-3P: diacylglycerol-3-phosphate; Tsr: chemotaxis receptor protein; PlsB: glycerol-3-phosphate acyltransferase; PlsC: 1-acylglycerol- phosphate acyltransferase

In addition to other organelles with membrane structures, the PM composed of phospholipid bilayers has piqued the interest of those focused on terpenoid research owing to its ability to store lipophilic molecules. PM engineering has received much attention particularly in the case of prokaryotic microorganisms that lack organelles. The combination of engineering membrane morphology and improving the membrane synthesis pathway could result in a 2.9-fold increase in β-carotene production in *Escherichia coli* [24]. Similarly, overexpression of the serine chemoreceptor Tsr in *E. coli* to wrinkle the inner membrane inward (also can expand the cell membrane) considerably increases the production of squalene synthesis, which is nearly 2.25 times that of the control strain (Fig. 2) [93]. Furthermore, localizing the enzymes close to their substrates within the PM can further improve catalytic efficiency. For instance, using membrane-anchoring peptides or proteins fused to β-carotene cleavage dioxygenase can increase the production of β-ionone [94, 95].

However, excessive hydrophobic molecules in the PM would reduce fluidity and stiffen the cell membrane, thereby causing cellular stress [43, 96, 97]. Increasing the proportion of unsaturated fatty acids in the PM could be a breakthrough in resolving this problem [98, 99].

## Transport system engineering strategies for effective synthesis of terpenoids

Higher titers may still be difficult to achieve if continuous terpenoid accumulation is noted in cells because of the limited storage capacity and their toxicity to organelles, proteins, DNA, and other biological processes [100, 101]. As a result, engineering efflux pumps to export terpenoids and thus reduce terpenoid cytotoxicity may be a promising solution [102, 103]. This would simultaneously assist in the recovery of the target products [104]. As typical efflux pumps, most ATP-binding cassette (ABC) transporters in the PM can identify and transport hydrophobic compounds [105–107].

**Fig. 3:**
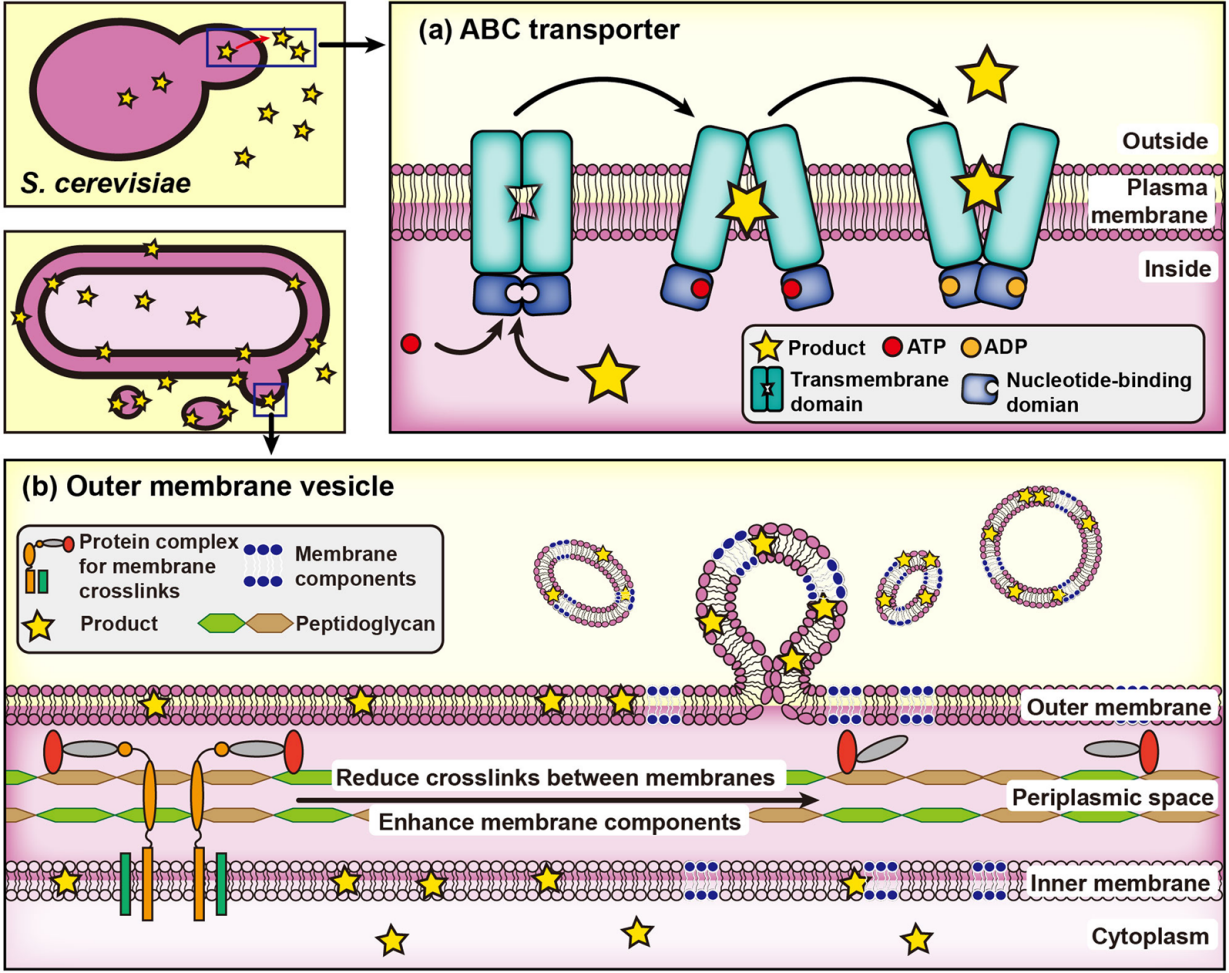
Schematic representation of transport system engineering strategies using *S. cerevisiae* and *E. coli* as an example. **a** Using ABC transporters in *S. cerevisiae* for terpenoid secretion. **b** Engineering *E. coli* cells to produce more outer membrane vesicles by reducing the crosslinks between the inner and outer membrane, and enhancing certain membrane components

Terpenoids and ABC transporters are correlated because they are both hydrophobic compounds. ABC transporter expression levels are higher in the strains that can efficiently synthesize terpenoids [108–110]. When ABC transporters are overexpressed, the synthesis of some terpenoids increases. For instance, overexpression of ABC transporter SNQ*2* resulted in a 4.04-fold and a 1.33-fold increase in β‑carotene secretion and intracellular production, respectively [111]. Moreover, increasing ATP supply or improving membrane fluidity can further increase the production of β-carotene, which is likely because ABC transporters are a class of membrane proteins driven by ATP (Fig. 3a) [112].

Besides a natural protein-based transport system, a novel artificial transport system based on membrane lipids, such as the outer membrane vesicles of *E. coli*, has initially been beneficial in terms of contributing to carrying and transporting terpenoids [113]. The amount of β-carotene secreted by the recombinant *E. coli* strain increased 53 times by promoting the formation of outer membrane vesicles and strengthening the synthesis of phosphatidylethanolamine to compensate for the loss of cell membrane components (caused by the formation of outer membrane vesicles), which indicated that this strategy provides a new direction for the extracellular transport of terpenoids (Fig. 3b) [113].

## Conclusion and perspectives

**Fig. 4:**
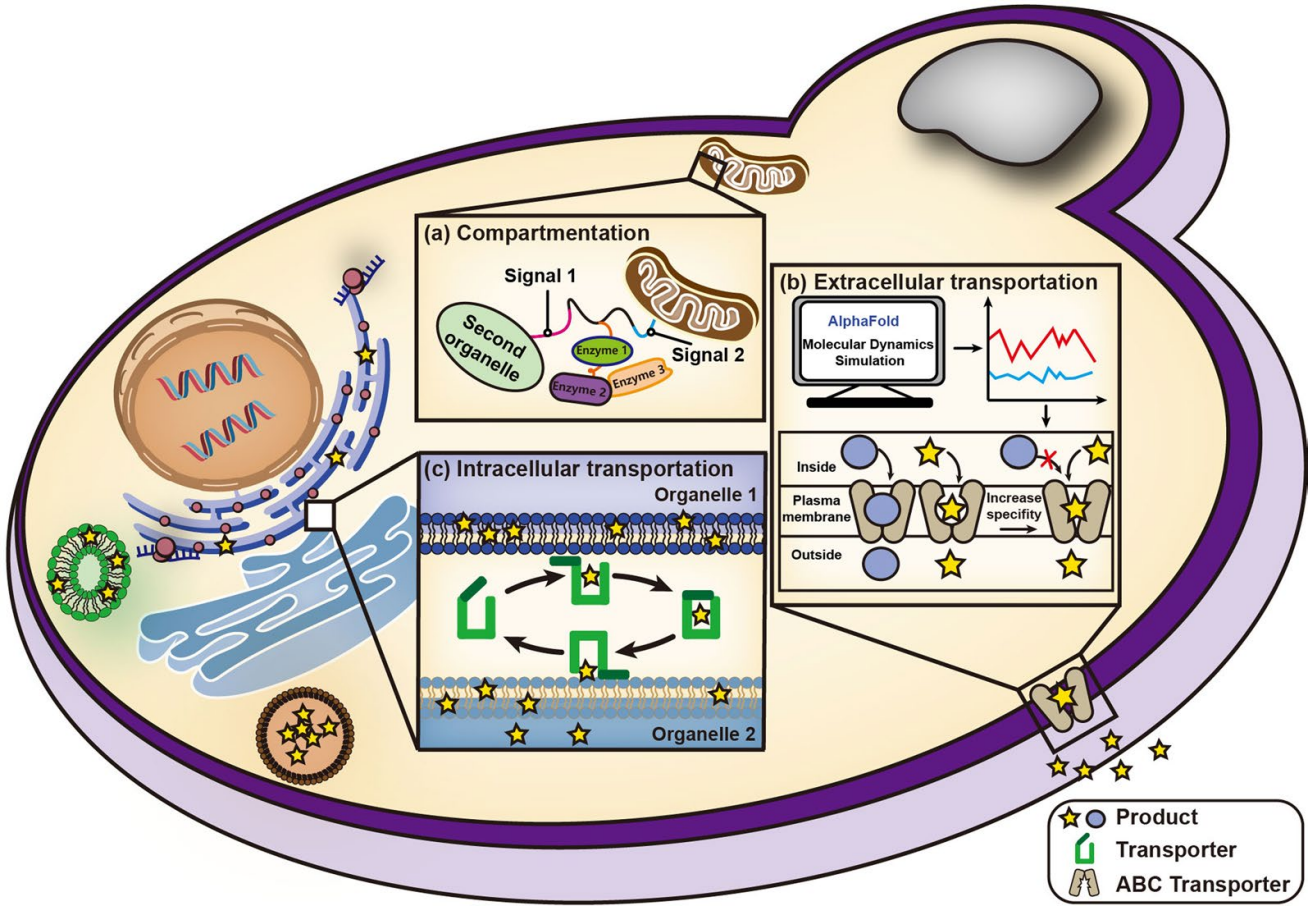
Future prospects of strategies that can effectively promote terpenoid synthesis using *S. cerevisiae* as an example. **a** Peptides with dual-localization may target enzyme complex to two different organelles. **b** Using methods like AlphaFold and molecular dynamics simulation for protein engineering to increase the substrate specificity of ABC transporters. **c** Mining terpenoid transporters to accelerate intracellular transportation

Rationally dividing the terpenoid synthesis pathway by anchoring key enzymes to appropriate organelles can increase the concentration of key enzymes and intermediates, which boosts the efficiency of compartmentalized pathway. Although the endomembrane system of certain eukaryotes has long been studied, new phenomena that provide a better insight into this system have emerged [114]. The interrelationships between organelles also deserve further investigation [115]. Simultaneously, additional research has provided a new theoretical basis for compartmentalization. For instance, the discovery of some dual-localization targeting signals may provide additional ideas and methods for enzyme localization (Fig. 4a) [84, 116–118]. As a result, the application of compartmentalization in terpenoid production remains a viable option. Another bottleneck that needs to be broken to increase terpenoid production is the limited storage capacity. However, it is unclear whether the expansion of organelle membranes will result in disorder between organelles or a stress response [119]. Apart from intracellular compartmentalization, taking advantage of constructing and optimizing the expression system and pathway module in parallel, intercellular compartmentalization can make full use of different biochemical characterizations of hosts and has also been applied in plant terpenoid production recently, such as oxygenated taxanes and strigolactones [120, 121]. Notably, this strategy requires that intermediate metabolites can cross cell membrane. Also, the difference in the doubling time of different hosts makes it particularly important to optimize culture conditions to synthesize more target products. Intercellular compartmentalization provides a promising strategy for complex plant terpenoids, especially when more functional plant-derived proteins are needed to be expressed.

While studies are now focusing more on using ABC transporters to secrete terpenoids to eliminate the toxicity caused by terpenoid accumulation, research that has focused on the mechanism underlying the transportation process is scarce. By analyzing the dynamic process of transportation using methods such as AlphaFold and molecular dynamics simulation, rational protein modification of transporters with poor substrate specificity can most likely be conducted to further improve terpenoid yield (Fig. 4b) [122]. Notably, the synthesis of terpenoids certainly passes through various organelles, such as ER, Golgi LD, and so on. To promote extracellular secretion, further enhancement of intracellular transportation can theoretically improve terpenoid synthesis to a considerable extent [123]. However, research on accelerating intracellular transportation is still in its infancy and more organelle transporters should be mined (Fig. 4c) [52].

## Data Availability

Not applicable.

## Abbreviations

ABC: ATP-binding cassette
CDW: Cell dry weight
DGAT: Diacylglycerol acyltransferases
DMAPP: Dimethylallyl diphosphate
ER: Endoplasmic reticulum
FPP: Farnesyl diphosphate
GgbAS: Glycyrrhiza glabra β-amyrin synthase
IPP: Isopentenyl diphosphate
LD: Lipid droplet
MTS: Mitochondrial targeting signals
MVA: Mevalonic acid
OSC: Oxidosqualene cyclase
PM: Plasma membrane
SE: Sterol ester
TAG: Triacylglycerol
